# Diabetic cardiomyopathy is associated with defective myocellular copper regulation and both defects are rectified by divalent copper chelation

**DOI:** 10.1186/1475-2840-13-100

**Published:** 2014-06-14

**Authors:** Shaoping Zhang, Hong Liu, Greeshma V Amarsingh, Carlos C H Cheung, Sebastian Hogl, Umayal Narayanan, Lin Zhang, Selina McHarg, Jingshu Xu, Deming Gong, John Kennedy, Bernard Barry, Yee Soon Choong, Anthony R J Phillips, Garth J S Cooper

**Affiliations:** 1 The School of Biological Sciences, Faculty of Science, University of Auckland, Auckland, New Zealand; 2 The Maurice Wilkins Centre for Molecular Biodiscovery, Faculty of Science, University of Auckland, Auckland, New Zealand; 3 Centre for Advanced Discovery and Experimental Therapeutics, Central Manchester University Hospitals NHS Foundation Trust, Manchester Academic Health Science Centre, and the Centre for Diabetes and Endocrinology, Institute of Human Development, Faculty of Medical and Human Sciences, University of Manchester, Manchester M13 9WL, UK; 4 National Isotope Centre, GNS Science, Gracefield, Wellington, New Zealand; 5 Department of Pharmacology, Medical Sciences Division, University of Oxford, Oxford, UK

**Keywords:** Diabetic cardiomyopathy, Copper-deficiency cardiomyopathy, Left-ventricular dysfunction, Myocellular copper, Copper transporters, Superoxide dismutase 1, Copper chaperones, Cu (II)-chelation, Copper metalation, Heart failure

## Abstract

**Background:**

Heart disease is the leading cause of death in diabetic patients, and defective copper metabolism may play important roles in the pathogenesis of diabetic cardiomyopathy (DCM). The present study sought to determine how myocardial copper status and key copper-proteins might become impaired by diabetes, and how they respond to treatment with the Cu (II)-selective chelator triethylenetetramine (TETA) in DCM.

**Methods:**

Experiments were performed in Wistar rats with streptozotocin (STZ)-induced diabetes with or without TETA treatment. Cardiac function was analyzed in isolated-perfused working hearts, and myocardial total copper content measured by particle-induced x-ray emission spectroscopy (PIXE) coupled with Rutherford backscattering spectrometry (RBS). Quantitative expression (mRNA and protein) and/or activity of key proteins that mediate LV-tissue-copper binding and transport, were analyzed by combined RT-qPCR, western blotting, immunofluorescence microscopy, and enzyme activity assays. Statistical analysis was performed using Student’s *t*-tests or ANOVA and *p*-values of < 0.05 have been considered significant.

**Results:**

Left-ventricular (LV) copper levels and function were severely depressed in rats following 16-weeks’ diabetes, but both were unexpectedly normalized 8-weeks after treatment with TETA was instituted. Localized myocardial copper deficiency was accompanied by decreased expression and increased polymerization of the copper-responsive transition-metal-binding metallothionein proteins (MT1/MT2), consistent with impaired anti-oxidant defences and elevated susceptibility to pro-oxidant stress. Levels of the high-affinity copper transporter-1 (CTR1) were depressed in diabetes, consistent with impaired membrane copper uptake, and were not modified by TETA which, contrastingly, renormalized myocardial copper and increased levels and cell-membrane localization of the low-affinity copper transporter-2 (CTR2). Diabetes also lowered indexes of intracellular (IC) copper delivery via the copper chaperone for superoxide dismutase (CCS) to its target cuproenzyme, superoxide dismutase-1 (SOD1): this pathway was rectified by TETA treatment, which normalized SOD1 activity with consequent bolstering of anti-oxidant defenses. Furthermore, diabetes depressed levels of additional intracellular copper-transporting proteins, including antioxidant-protein-1 (ATOX1) and copper-transporting-ATPase-2 (ATP7B), whereas TETA elevated copper-transporting-ATPase-1 (ATP7A).

**Conclusions:**

Myocardial copper deficiency and defective cellular copper transport/trafficking are revealed as key molecular defects underlying LV impairment in diabetes, and TETA-mediated restoration of copper regulation provides a potential new class of therapeutic molecules for DCM.

## Background

Cardiovascular disease is the leading cause of disability and death in patients with diabetes mellitus [1,2], in whom it is frequently accompanied by impaired LV function and heart failure [3,4]. Hyperglycemia is a major factor for the development of diabetic cardiomyopathy (DCM) which contributes substantially to morbidity and mortality [5-7]. DCM causes numerous pathological changes, for example myocellular hypertrophy, interstitial fibrosis and defective fuel utilization, accompanied by damage to myocellular organelles including the plasma membrane, contractile apparatus, mitochondria, and sarcoplasmic reticulum: these defects cooperate to cause impaired systolic and diastolic function and frequently progress to overt heart failure [7-12]. The molecular mechanisms by which these defects occur are poorly understood and currently there is no directly effective treatment for DCM [3,7].

Pathogenetic mechanisms that cause tissue damage in diabetes may reflect molecular defects that lead to increased cellular production of superoxide anion (O_2_^●-^) in affected tissues [13,14]: examples are excessive O_2_^●-^ production through mitochondrial dysfunction [15] coupled with diminished O_2_^●-^ clearance through impaired SOD activity (reviewed in [16]). In addition, alterations in ion homeostasis have also been implicated in the pathogenesis of DCM [7,17]. For example, decreased myocellular Ca^2+^ efflux through the Na^+^-Ca^2+^ exchanger (NCX) and Ca^2+^-ATPase (SERCA) pump systems, has been linked to defects in cardiac energy metabolism and contractile function [17,18]; however, impaired calcium homeostasis may not explain the contractile deficit in DCM [9,10]. Altered myocellular [K^+^]_IC_ and [Na^+^]_IC_ may also contribute to the impaired cardiac function and energy-inefficient metabolism in DCM [19,20].

Both copper deficiency [21,22] and copper excess states [23] can cause elevated oxidative stress and impaired antioxidant defenses [24,25]. Copper deficiency can cause neurodegeneration [26], and hematological and cardiovascular disorders [22], whereas copper overload may be accompanied by hepatic and neurological diseases [27,28]. Copper homeostasis is coordinated by several regulatory protein chaperones, through which it is delivered to specific subcellular compartments and/or copper-requiring proteins without releasing free copper atoms that could otherwise damage cells and tissues [29,30]: examples include the copper chaperone for superoxide dismutase (CCS) for SOD1, and the antioxidant 1 copper chaperone (ATOX1) for ATP7A and ATP7B. Disturbances in copper homeostasis have pronounced deleterious effects on many bodily functions, such as those observed in Menkes’ or Wilson’s diseases, which are caused by mutations in the genes encoding ATP7A and ATP7B respectively [27,31]. In addition, copper is a key cofactor for many important enzymes such as Cu/Zn-SOD (SOD1) [32], EC-SOD (SOD3) [33], cytochrome c oxidase (COX) [34], and ceruloplasmin/ferroxidase [35], whereof deficient activities have been implicated in the causation of several disease states [36-38]. It has also been shown that localized myocardial copper deficiency, caused by the heart-specific knockout of Ctr1 in mice, causes severe cardiomyopathy [39], as do genetically-mediated defects in humans and rodents of the *SCO2* gene, which encodes a chaperone protein necessary for copper metalation of the CuA site on the COII subunit of COX [40,41]. These observations provide key evidence linking myocardial copper deficiency and impaired copper metalation to the causation of cardiomyopathy.

Copper deficiency causes cardiomyopathy in several animal species [42,43], wherein its pathobiology closely resembles that of DCM [24,42,43]. However, indexes of systemic copper regulation differ markedly between the two conditions. Animals with cardiomyopathy caused by insufficient copper intake exhibit clear signs of *systemic copper deficiency*, including hypocupremia, hypoceruloplasminemia, anemia and neutropenia, and deficient hepatic copper levels [44], all of which can be alleviated by copper replacement. By contrast, diabetic animals and patients with DCM show signs of *systemic copper excess* with elevations in urinary copper and copper balance, normal or elevated plasma copper and ceruloplasmin levels [8,16,45,46], and markedly elevated hepatic and renal copper levels [46,47]. These observations indicate that impaired copper metabolism occurs in diabetes, and that defective copper regulation could play specific roles in the pathogenesis and progression of the diabetic complications.

It has previously been shown that Cu (II) chelation with triethylenetetramine (TETA) restores indexes of systemic copper homeostasis and LV mass in diabetic patients with LV hypertrophy [48], and improves cardiac structure and function in rat models of diabetes [8,10,49,50]. The current study was designed to investigate the effects of diabetes on copper status and indexes of myocellular copper transport/trafficking, and their potential contribution to the development of heart disease in a widely-accepted rat model of DCM. We also investigated the molecular mechanisms by which TETA treatment ameliorates diabetes-induced dysregulation of cardiac copper homeostasis, which could contribute to observed TETA-mediated improvement in cardiac function. We compared myocardial expression (mRNA and protein) of key components of the cellular copper-transport pathways, which coordinate the regulation of copper homeostasis in cardiac LV tissues, in groups of non-diabetic control, diabetic, and TETA-treated-diabetic animals; we also undertook some studies in TETA-treated non-diabetic animals for comparative purposes (Table 1). We also examined the effects of TETA treatment on the expression and cellular translocation of copper-transporter proteins and copper-enzymes. In addition, we measured changes in LV-copper content and its response to TETA treatment, in relation to alterations in the expression/activity of copper-regulatory proteins in rats with DCM.

**Table 1:** **Relevant experimental group characteristics and hemodynamic parameters in the isolated perfused hearts of non**-**diabetic control**, **TETA**-**treated control**, **diabetic**, **and TETA**-**treated diabetic rats**

**Variable**	**Control**	**TETA**-**control**	**Diabetic**	**TETA**-**diabetic**
Strain	Wistar	Wistar	Wistar	Wistar
Sex	Male	Male	Male	Male
Number	9	9	9	9
Age at enrolment (weeks)	6-7	6-7	6-7	6-7
Age when studied (weeks)	22-23	22-23	22-23	22-23
Body weight (g)	573 ± 16	562 ± 17	220 ± 9*	290 ± 21*
Blood glucose (mM)	5.8 ± 0.20	5.1 ± 0.15	29.8 ± 0.65*	27.0 ± 1.20*
Heart weight (g)	1.58 ± 0.04	1.63 ± 0.05	1.03 ± 0.06*	1.18 ± 0.09*
Heart-weight/Body-weight (×10^−3^)	2.76 ± 0.01	2.77 ± 0.13	4.66 ± 0.13*	4.08 ± 0.17*^#^
Cardiac output (ml/min)	79.2 ± 3.2	75.0 ± 3.5	53.3 ± 8.1*	78.0 ± 4.0^#^
LV + dP/dt max (mmHg/s)	4506 ± 549	4667 ± 417	2249 ± 162*	4082 ± 196^#^
LV -dP/dt min (mmHg/s)	−4384 ± 453	−4245 ± 413	−1952 ± 144*	−3366 ± 125^#^

Measurements of steady-state cardiac function were made at 20 cm-H_2_O preload and 75 mmHg afterload of hearts paced at 300 beats/min. Values are means ± SEM. Data were analyzed using two-way ANOVA with *post*-*hoc* Tukey’s tests: *P < 0.05 vs control; ^#^*P* < 0.05 vs diabetic.

## Methods

### Animal studies

Protocols for the induction of diabetes in rats and TETA treatment were as previously described [8,49]. Diabetes was induced by a single intravenous injection of STZ (55 mg/kg body-weight; Sigma) into the tail-veins of adult male Wistar rats (6–7 weeks of age; 220–250 g); control animals received a saline injection instead of STZ. TETA was administered via the drinking water (20 mg/day per rat, Fluka), beginning at 8 weeks after saline or STZ injection. LV tissues from each treatment group (non-diabetic control, diabetic, TETA treated-control and TETA treated-diabetic) were collected after 8-weeks’ treatment. All experimental protocols were approved by the Animal Ethics Committee of the University of Auckland. The study was performed according to the ‘Guide for the Care and Use of Laboratory Animals’ [51], and this manuscript is consistent with the ‘ARRIVE guidelines for the reporting of animal research’ [52].

### Choice of TETA dosage

The dosage used here was based on those employed in known clinical applications of TETA (as TETA dihydrochloride or trientine) in the treatment of patients with Wilson’s disease, and for the experimental therapy of diabetes [10]. In brief, dosages employed for the treatment of Wilson’s disease in adults typically vary from 750–2000 mg/day (equivalent to ~11-29 mg/kg-day in 70-kg adults) [53]. Here, we administered TETA dihydrochloride in the drinking water to diabetic rats at 20 mg/day (equivalent to ~68 mg/kg-day of trientine in 250-g rats). This dosage is supported by our published dose-rising phase-1 clinical trial, where we showed that dosages of 1200 and 3600 mg/day (equivalent to ~17 and 51 mg/kg-day in 70-kg adults) were effective and well tolerated in healthy adult human volunteers [54], and also by our phase-2 trial where 1200 mg/day of trientine (~17 mg/kg-day in 70-kg adults) administered for 12 months markedly improved LV mass in T2D patients with LV hypertrophy [48].

### Choice and validation of animal model

We have extensively validated the STZ-based model we chose to employ here by showing that these diabetic rats respond both to untreated diabetes and following TETA treatment, in ways that closely reflect responses in patients with T2D [8,10,16,45,48,49,55]. To date, we have shown that TETA treatment exerts substantial effects to restore LV mass in patients with LV hypertrophy [48], similar to its effects in rats [8,49], and that the dosage-responsiveness of TETA-mediated removal of copper from the body is similar in rats [8] and human volunteers [54,56]. These findings provide strong support for our use of this model for the current studies.

### Measurement of *ex*-*vivo* cardiac function

We measured cardiac function in isolated, *ex*-*vivo* perfused working hearts, as previously detailed [8,49]. On the experimental day, rats were anesthetized (isoflurane), heparinized (1,000 IU/kg i.v.), and hearts excised and immersed in 4°C Krebs-Henseleit bicarbonate buffer (KHB). Retrograde (Langendorff) perfusion was established (KHB, 37°C, gassed with O_2_:CO_2_ 95:5 (vol/vol). Working-mode perfusion was then established (preload, 10 cmH_2_O; afterload, 55.9 mmHg) with pacing (300 bpm; Digitimer). Intra-chamber LV pressure (SP855; AD Instruments), aortic pressure (PX23XL, Stratham Gould), and aortic (Transonic T206) and coronary flows were measured; pressure and flow data were recorded (Powerlab16s, ADI); and the maximum rate of ventricular pressure development (+dP_LV_/dt) and minimum rate of relaxation (−dP_LV_/dt) were derived. Atrial filling pressure was decreased (to 5 cmH_2_O) and then increased (in seven equal steps of 2.5 cmH_2_O to 20 cmH_2_O [final]), and 1-min averages were extracted. Filling pressure was then fixed at 10 cmH_2_O, and afterload at 75 mmHg.

### Measurement of tissue copper content

Copper concentrations were determined in dry *ex vivo* LV-tissue by using a reference method, PIXE coupled with RBS [57]. The calibration, measurements, and limits of detection were based on the areas of the K_α_ x-ray peaks as measured by the software package GUPIX Elemental, and concentrations were extracted from PIXE spectra using GUPIX software (University of Guelph, ON, Canada), as previously described [10,46].

### Measurement of mRNA by RT-qPCR

Here, RT-qPCR was performed according to ‘The Minimum Information for Publication of Quantitative Real-Time PCR Experiments (MIQE)’ guidelines [58]. Total RNA was extracted from rat LV tissues that had previously been preserved (RNAlater, Ambion), by using an RNeasy mini kit (Qiagen), and was reverse-transcribed into cDNA using a first-strand cDNA synthetase kit (Roche Diagnostics). Real-time qPCR protocols were designed based on the MIQE guidelines and performed using the SYBR Green technology on a LightCycler rapid thermal cycler (Roche Diagnostics), in accordance with the manufacturer’s instructions. Quantification results for mRNA levels for each target gene were normalized to the geometric mean of the expression of three optimal reference genes for the study of gene expression in cardiac tissue (*Rpl13a*, *Tbp* and *Ndc1*). Evaluation of reference genes and PCR primers employed is described in Additional file 1.

### Western blot analysis

Protein extracts from LV tissues were prepared by adding ice-cold RIPA buffer supplemented with a protease inhibitor cocktail (Roche, Basle, Switzerland), and homogenised using a TissueLyser II (Qiagen). After centrifugation (20 min, 16,500 × *g*, 4°C), protein concentrations of supernatants were determined by BCA assay (Thermo Scientific). Western blotting was performed as previously described [9]. Briefly, 25 μg of each sample was loaded onto a 12% Bis-Tris-polyacrylamide gel (Invitrogen) and transferred to nitrocellulose membranes. Blots were first probed with specific antibodies as follows: CTR1 (sc-66847, Santa Cruz Biotechnology Inc); CTR2 (sc-104852, Santa Cruz Biotechnology Inc); MT1/2 (sc-11377, Santa Cruz Biotechnology Inc), and then incubated with an HRP-conjugated IgG, (dilution 1:10,000, Jackson Laboratories). Specific protein bands were detected by using an enhanced chemiluminescent substrate (ECL Plus, GE Healthcare, Buckinghamshire, UK) and imaged using an LAS 3000 image reader (Fuji Photo Film Co. Ltd, Tokyo, Japan). Ponceau-S staining was used as the reference for loading controls, and band intensities were measured using MultiGauge software v2.0 (Fuji Photo Film).

### Cu/Zn-SOD1 ELISA

LV tissues were weighed and minced into small pieces before homogenization in phosphate-buffered saline (pH 7.2; 20 μl/mg tissue) using a TissueLyser II (Qiagen). The resulting suspension was subjected to two freeze-thaw cycles and then centrifuged (5 min, 5000 × *g*, 4°C). Thereafter, supernatants were removed and total-protein concentrations determined by bicinchoninic acid (BCA) assay (Thermo Scientific). SOD1 concentrations were measured using an ELISA kit according to the manufacturer’s protocol (USCN Life Science; Wuhan, Hubei, PRC) by spectrophotometry (450 nm, microplate reader; SpectraMAX 340, Molecular Devices; Sunnyvale, CA), and the concentration of SOD1 (ng/mL) in the sample supernatants calculated by comparing the O.D. of the samples to a standard curve. Results were then converted to ng/mg total protein and expressed as concentrations relative to the corresponding control group.

### Cu/Zn-SOD1 activity assay

LV tissues were homogenized in ice-cold 0.1 M Tris/HCl, pH 7.4 containing 0.5% Triton X-100, 5 mM mercaptoethanol and 0.1 mg/ml PMSF using a TissueLyser II (Qiagen). The crude tissue homogenates were then centrifuged (14,000×*g*, 5 min, 4°C) and the supernatants collected: these contained total SOD activity from combined cytosolic and mitochondrial sources. Following incubation with 0.4 vol of a solution containing ethanol and chloroform (25:15 vol/vol) to inactivate the mitochondrial SOD2 [59], mixtures were centrifuged at 5000 × g for 15 min and aliquots of the supernatant used to measure protein concentration by BCA assay, and Cu/Zn-SOD activity using the Superoxide Dismutase Activity Assay kit (Abcam). The latter works by measuring the inhibition of the reduction of a water-soluble tetrazolium salt (WST-1) which produces a water-soluble formazan dye upon reduction by O_2_^●-^ radicals, which are generated by xanthine oxidase and inhibited by SOD. One unit of SOD activity is the amount necessary to inhibit the xanthine oxidation by 50%. The IC_50_ (50% inhibition activity of SOD) was detected by a colorimetric method at 450 nm using an absorbance microplate reader (SpectraMAX 340, Molecular Devices) and the results expressed per mg of total protein.

### Immunofluorescence analysis of LV free-wall tissue

Immunofluorescent staining was performed using standard techniques as described. Briefly, 6-μm LV sections frozen in OCT were cut, fixed in acetone, and blocked with 5% (vol/vol) normal donkey serum (NDS) in PBS. They were then incubated overnight at 4°C with primary antibody in 5% NDS (vol/vol). Primary antibodies used in these experiments were: anti-CTR1 (at 1:200 vol/vol final dilution, Abnova); anti-CTR2 (1:50, Santa Cruz); MT1/2 (1:100, Abcam); CCS (1:200, Novus Biologicals); ATOX1 (1:100, Santa Cruz); ATP7A (1:250, Abcam), ATP7B (1:150, Novus Biologicals), and N-cadherin (1:50, Abcam). Following 3 × washes with PBS, sections were incubated with appropriate fluorescently-labeled (−FITC or -Rhodamine Red, as required) donkey anti-rabbit/mouse/chicken IgG secondary antibodies (Jackson ImmunoResearch). After incubation, sections were washed and incubated overnight with WGA-Oregon Green 488 (1:5000; Molecular Probes) in PBS at 4°C. Finally, sections were washed again and mounted with Prolong Gold anti-fade reagent with DAPI (Invitrogen). The immunofluorescent images were captured at 40×, 60× or 100× magnifications using a confocal fluorescent microscope (Olympus FV1000, Fuji).

The fluorescently-labeled signals from each image were processed and quantified using a custom-written IDL analysis program based on signal-thresholding, as previously described [9,10]. Briefly, the original image was masked to exclude non-labeled areas. The threshold for quantifying fluorescent labeling was then established, based on information in the masked image’s histogram compared with the original image. The criteria for the thresholding process were maintained equivalent across all experimental groups. Results were expressed as percentages of corresponding total cross-sectional areas. To avoid any possible confounding effect of orientation and origin of the muscle fibers on the signal quantification, at least five transverse optical-sectional images from each section of the LV-myocardium, two sections from each animal, and four animals per group, were analyzed.

### Statistical analysis

Data have been expressed as mean ± SEM unless stated otherwise. Significance of between-group differences was determined by unpaired Student’s *t*-tests, one-way ANOVA, or two-way ANOVA, as appropriate. The primary *a priori* null hypothesis in each contrast was that there was no difference between signals from TETA-treated diabetic and untreated diabetic states. A second between-group comparison was performed in each case, with the *a priori* null hypothesis being that there was no difference between signals from control and diabetic animals: this latter contrast was included to ensure that effects of diabetes were present as a starting condition for comparison with TETA-treated samples. Where stated in the figure legends, correction for multiple-comparisons has been applied. All contrasts were two-tailed, and values of *P* < 0.05 have been considered significant.

## Results

### Diabetes caused myocardial copper deficiency that was rectified by TETA treatment

**Figure 1:**
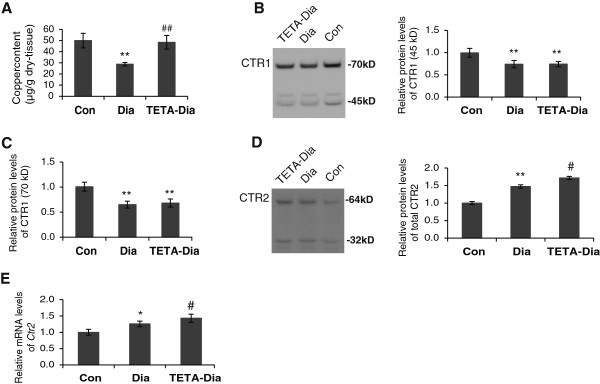
**Measurement of total myocardial copper content**, **and mRNA and protein levels of copper**-**uptake transporters CTR1 and CTR2 in LV-tissue from control**, **diabetic and TETA**-**treated diabetic rats. A**: Total myocardial copper concentrations (μg/g dry LV tissue) measured by PIXE/RBS in LV sections. Values are means ± SEM; significance of differences has been analyzed by one-way ANOVA with Bonferroni’s multiple-comparisons test: **P < 0.01, diabetic vs. control; ^##^*P* < 0.01, TETA-diabetic vs. diabetic: n = 7/group. **B** and **C**: Representative Western blots for CTR1. The intensities of the CTR1 bands (~45 kD and ~70 kD, respectively) normalized to Ponceau-S-stained bands are depicted graphically. **D**: Representative Western blot of CTR2. The total intensities of the CTR2 protein bands (~32 kD and ~64 kD) relative to Ponceau-S-stained bands are depicted graphically. **E**: RT-qPCR analysis of *Ctr2* mRNA levels. Results were normalized to a robust normalizer (geometric mean of mRNA levels of *Rpl13a*, *Tbp* and *Ndc*). All data are means ± SEM and presented as relative to the respective controls, which were set at 1: *P < 0.05, **P < 0.01 vs. control and ^#^*P* < 0.05 vs. diabetic: n = 7/group **(B**, **C**, **D)**, and n = 9/group **(E)**.

LV-copper levels were markedly deficient (decreased to ~50% of normal) after 16-weeks’ untreated diabetes. Strikingly, LV-copper levels were fully restored to normal (control) concentrations when TETA treatment was instituted after 8-weeks’ diabetes and maintained for a further 8 weeks (Figure 1A): this effect was accompanied by substantial improvements in cardiac function, including in cardiac output, and indexes of systolic and diastolic function (Table 1). However, TETA treatment did not change any index of cardiac function in non-diabetic control rats (Table 1), consistent with previous reports [8,60].

### TETA treatment accentuated diabetes-evoked up-regulation of CTR2, whereas it did not modify diabetes-elicited lowering of Ctr1 expression

**Figure 2:**
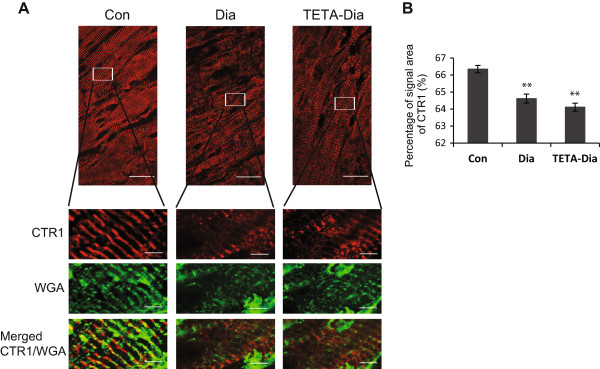
**Immunofluorescent analysis of CTR1 localization in LV sections from control**, **diabetic and TETA**-**treated diabetic rats. A**: Representative LV-wall longitudinal sections (100 ×-objective) labeled with anti-CTR1 (*red*) and WGA (*green*): n = 40 sectional images/group: scale bar = 20 μm. For further magnified images, scale bar = 4 μm. **B**: Quantitative analysis of immunofluorescent-signal area for CTR1. Confocal images (40 ×-objective) were analyzed using a custom-written IDL analysis program based on signal-thresholding. Results are expressed as mean of the percentages of corresponding cross-sectional areas: **P < 0.01 vs. control: at least 40 sectional images/group were analyzed.

Immunoblotting for the high-affinity copper transporter, CTR1, indicated the presence of dimeric (~45 kD) and trimeric (~75 kD) forms, both of which were diminished in diabetic LV myocardium as compared to control (Figure 1B and 1C). TETA treatment did not restore CTR1 expression (Figure 1B and 1C), and *Ctr1* mRNA levels were unchanged in all treatment groups: furthermore, CTR1 protein levels were also unchanged in TETA-treated controls (data not shown).Confocal imaging was used to further investigate the role of CTR1 in copper-deficient diabetic LV. CTR1 was found to co-localize predominantly with the myocardial T-tubule membrane system in control LV (Figure 2A); however, CTR1 staining appeared to be diminished and irregular in diabetic LV, consistent with possible disruption of the T-tubules themselves. TETA-treated diabetic tissues may have a slightly more organized CTR1 staining localization as compared to untreated diabetes, although this effect is subtle. In addition, quantitative immunofluorescent analysis of CTR1 confirmed the diminished staining intensity in both diabetic and TETA-treated diabetic LV (Figure 2B). These results are consistent with CTR1 immunoblotting data, and cumulatively support the down-regulation of CTR1 as being a potential mechanism of impaired copper uptake and subsequent copper deficiency in diabetic LV.

**Figure 3:**
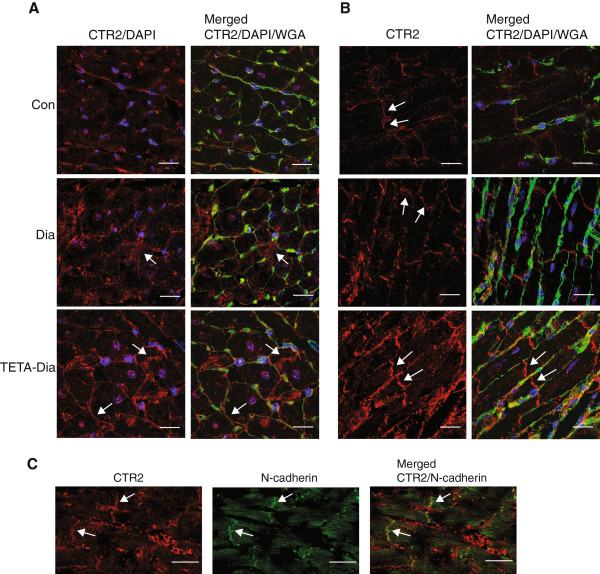
**Immunofluorescent micrographs of CTR2 localization in sections of the LV free**-**wall from control**, **diabetic and TETA**-**treated diabetic rats. A**: Representative LV wall cross-sections (100 ×-objective) labeled with anti-CTR2 antibody (RRX *red*). Arrows indicate ‘vesicle-like’ structures adjacent to the sarcolemmal membrane. **B**: Representative longitudinal sections of LV-wall (100 ×-objective) labeled with anti-CTR2 antibody (*red*). Arrows indicate intercalated disks. All sections in A and B were co-stained for sarcolemmal membranes (*green*) and nuclei (*blue*) with WGA-Oregon Green 488 and DAPI, respectively: n = 40 sectional images/group: scale bar = 20 μm. **C**: Representative double-immunofluorescent labeling of CTR2 and N-cadherin in longitudinal LV sections (60×-objective). Arrows indicate co-localization at the intercalated disk: n = 10 sectional images/group: scale bar = 30 μm.

As TETA treatment did not restore CTR1 protein expression in diabetes, it seemed unlikely that this could be a mechanism by which TETA restores LV-copper levels. Interestingly, immunoblotting for the lower affinity-copper transporter, CTR2, showed increased expression of both dimeric (~32kD) and tetrameric (~64 kD) CTR2 forms in diabetic LV (Figure 1D). CTR2 is known to form functional homo-multimers and the specificity of bands was verified here by antibody blocking (data not shown). Furthermore, *Ctr2* mRNA was also increased in diabetic LV as compared to control (Figure 1E). Further studies showed that, unlike CTR1, TETA treatment significantly elevated CTR2 expression, although rather than merely restoring levels, it further increased both *Ctr2* mRNA and protein expression as compared to untreated diabetic LV (Figure 1D and 1E), whereas by contrast, it has no effects on CTR2 expression in non-diabetic controls (data not shown). These results are consistent with an increase of copper uptake via CTR2 as a key part of the mechanism by which TETA restores myocardial copper content.We employed confocal imaging to further examine the role of CTR2. Transverse sections of LV showed CTR2 localized to the nucleus and intracellular vesicles of myocytes, along with subtle staining of the sarcolemmal membrane in controls. This staining pattern was more intense in diabetic LV, particularly in the intracellular small vesicles, from which copper is known to translocate to the cytosol (Figure 3A). This response correlates well to enhanced CTR2 protein expression, which could represent an endogenous, self-compensating response towards maintenance of cytosolic copper in diabetes. Interestingly, LV from TETA-treated animals showed further increases in CTR2 staining at or near the outer sarcolemmal membrane: increased expression of CTR2 in this location could well mediate enhanced uptake of extracellular copper into cardiomyocytes. Moreover, longitudinal LV sections confirmed enhanced staining of CTR2 at the cardiomyocyte periphery, particularly at the intercalated discs of TETA-treated diabetic LV (as compared to control and untreated-diabetic LV) (Figure 3B), consistent with increased transport of copper between neighboring myocardial cells. Co-staining of N-cadherin with anti-CTR2 confirmed the intercalated disc localization (Figure 3C). In addition, the enhanced localization of CTR2 at the outer sarcolemmal membrane in TETA-treated LV is a candidate mechanism by which TETA could enhance copper uptake and restore LV copper content.

### Diabetes lowered the expression of MT1 and MT2 in LV myocardium

**Figure 4:**
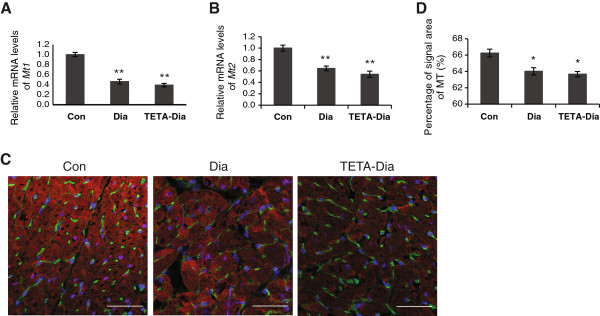
**mRNA levels and percentages of protein signal**-**areas corresponding to MT1**/**2 in LV tissues from control**, **diabetic and TETA**-**treated diabetic rats. A** and **B**: RT-qPCR analysis of *Mt1* and *Mt2* mRNA levels. All data are means ± SEM and presented relative to their respective controls, which were set at 1: n = 9/group. **C**: Representative confocal images of immunofluorescent-stained cross-sections of LV (40 ×-objective) labeled with anti-MT1/2 (*red*), WGA-Oregon Green 488 for sarcolemmal membrane (*green*), and DAPI for nuclei (*blue*). At least five images/section, two sections/animal, and four animals/group were examined: scale bar = 50 μm. **D**: Quantification of immunofluorescent-labeled myocardial signals areas for MT1/2. Results are means ± SEM and presented as the percentages of corresponding cross-sectional areas. At least five transverse optical sectional images/section, two sections/animal and four animals/group were analyzed: n = 40 sectional images/group: *P < 0.05, **P < 0.01 vs. control.

Deficient myocardial-copper levels could be associated with altered expression or function of intracellular copper-regulating proteins. We therefore examined the expression of the copper-binding proteins, MT1 and MT2, which act to maintain low levels of free transition-metal ions (particularly Cu and Zn) within cells, and play essential roles in copper-ion detoxification [61]. RT-qPCR demonstrated a significant reduction in mRNA levels of *Mt1* and *Mt2* in diabetic LV compared with control tissue (Figure 4A and 4B), and TETA treatment did not reverse these changes. The lower levels of *Mt1* and *Mt2* mRNAs observed in diabetic LV tissue are consistent with the observed decreases in myocardial copper content, since expression of *Mt1* and *Mt2* is known to be primarily regulated by intracellular metal concentrations at the level of transcription [61].Confocal micrographs of control LV stained for combined MT1/2 showed intense sarcoplasmic and nuclear localization, with lower-intensity staining of the sarcolemmal membrane (Figure 4C). MT1/2 staining intensity was notably diminished in both untreated and TETA-treated diabetic cardiomyocytes, with some stronger staining persisting near the sarcolemmal surface. Quantification of the fluorescent signal area confirmed diminished staining intensity of MT1/2 in diabetic and TETA-treated diabetic LV as compared to control (Figure 4D). Furthermore, no notable change in the localization of MT1/2 signal intensity was detected in diabetic or corresponding areas of TETA-treated LV. Together, these results are consistent with decreased capacity of cytosolic copper binding and thus decreased protection against copper toxicity in diabetes.

### Increased polymerization of MT in diabetic rats was partially ameliorated by TETA treatment

**Figure 5:**
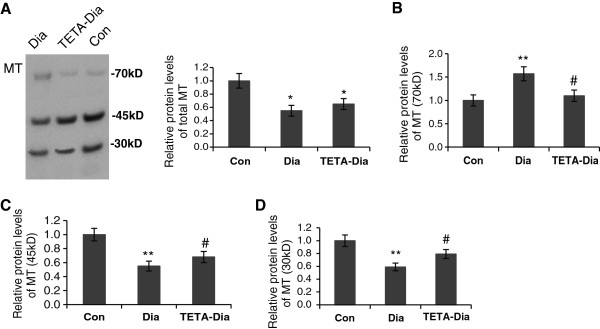
**Representative Western blots of LV extracts probed for anti**-**MT1**/**2.** The intensities of the immunoreactive MT1/2 protein bands **(A**, **B**, **C**, **D** for total, ~70 kD, ~45 kD and ~ 30 kD polymerized forms of MT1/2, respectively) normalized to corresponding Ponceau-S-stained bands and depicted graphically. Quantitative data are means ± SEM and presented as relative to the non-diabetic controls, which were set at 1: *P < 0.05, **P < 0.01 vs. control, and ^#^*P* < 0.05 vs. diabetic: n = 7/group.

Oxidation of the sulfhydryl groups in MT can lead to their polymerization and altered copper-binding properties. Western blotting of LV did not detect monomeric MT1/2, but instead several higher molecular-weight bands of ~30, 45 and 70 kD in all 3 treatment groups (Figure 5A) (specificity of MT bands was verified by antibody blocking studies, data not shown). Combined densitometry of all 3 polymerized MT bands indicated a reduction of ~34% expression in diabetes which TETA treatment did not significantly alter (Figure 5A), consistent with the lowering in the MT immunofluorescent signal shown above.However, densitometry of the respective MT band sizes revealed that ~70 kD MT was significantly increased, by ~50%, in diabetic LV tissue (Figure 5B), whereas the ~45kD and ~30 kD bands were significantly reduced, by 48% and 40% respectively, in diabetic LV as compared to control (Figure 5C and 5D). Interestingly, TETA treatment partially corrected diabetes-induced changes in the expression of all 3 polymerized MT bands (Figure 5B, 5C and 5D). These results provide clear evidence that TETA treatment could result in decreased polymerization of MT, which is consistent with decreased oxidative stress in the TETA-treated diabetic heart.

### TETA rectified diabetes-mediated defects in CCS and SOD1

**Figure 6:**
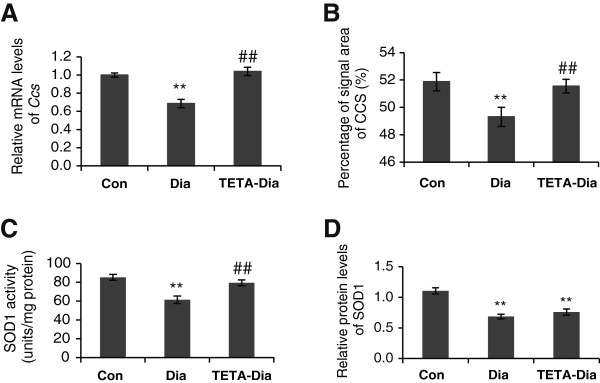
**Expression and activity analyses of CCS and SOD1 in LV tissues from control**, **diabetic**, **and TETA**-**treated diabetic rats. A**: RT-qPCR analysis of *Ccs* mRNA levels. Data are means ± SEM and presented relative to the respective controls which were set at 1: **P < 0.01, vs. control; ^##^*P* < 0.01 vs. diabetic: n = 9/group. **B**: Quantitative analysis of immunofluorescent signal area for CCS. Results are expressed as means of the percentages of corresponding cross-sectional areas. At least 40 sectional images/group were analyzed: **P < 0.01 vs. control; ^##^P < 0.01 vs. diabetic. **C**: Measurement of SOD1 activity by monitoring xanthine oxidase activity, which is inhibited by SOD1. The IC_50_ (50% inhibition activity of SOD) was detected by a colorimetric method at 450 nm and the results calculated as units/mg of total protein (based on a unit of SOD activity as the amount necessary to inhibit xanthine oxidation by 50%): **P < 0.01 vs. control; ^##^*P* < 0.01 vs. diabetic: n = 9/group. **D**: Measurement of protein levels of SOD1 in LV extracts by ELISA. Results were calculated as ng/mg total protein and are presented as the means of relative concentrations related to the control group, which was set at 1: **P < 0.01 control: n = 9/group.

CCS delivers copper to the apoenzyme of SOD1 to yield the active holoenzyme. RT-qPCR demonstrated *Ccs* mRNA levels were significantly down-regulated, by ~30%, in diabetic LV compared with control tissue (Figure 6A). Interestingly, TETA treatment reversed this decrease in mRNA expression to levels similar to non-diabetic control values. Furthermore, quantitative immunofluorescent analysis showed that diabetic LV had decreased CCS-specific signal areas, which were normalized by TETA treatment (Figure 6B). These results show that transcriptional and translational dysregulation of CCS occurs in diabetes and is rectified by TETA treatment.

As the down-regulation of CCS in diabetes could well impair its ability to deliver copper to SOD1, we investigated whether SOD1 activity was altered in diabetes. Indeed, SOD1 activity was significantly decreased in diabetic LV, whilst TETA-treated diabetic tissue showed restoration of SOD1 activity (Figure 6C). ELISA of SOD1 protein levels showed a significant decrease in diabetic myocardium compared to control values, which TETA treatment did not restore (Figure 6D), although no detectable between-group differences in *Sod1* mRNA levels were present (data not shown). These results indicate that SOD1 is subject to post-transcriptional down-regulation in diabetic LV tissue. We also found that TETA had no detectable effects on SOD1 expression in diabetic LV tissue (Figure 6D). These results, when taken together, indicate that decreased expression of SOD1 and CCS contribute to the deficiency in LV-myocardial SOD1 activity in diabetes. They also indicate that TETA treatment activates SOD1 by increasing the supply of copper for incorporation into the enzyme via improved CCS function, without changing the expression of SOD1 protein itself.

### Diabetes and TETA treatment evoked different responses in the regulation of the copper-transporting ATPases ATP7A and ATP7B

**Figure 7:**
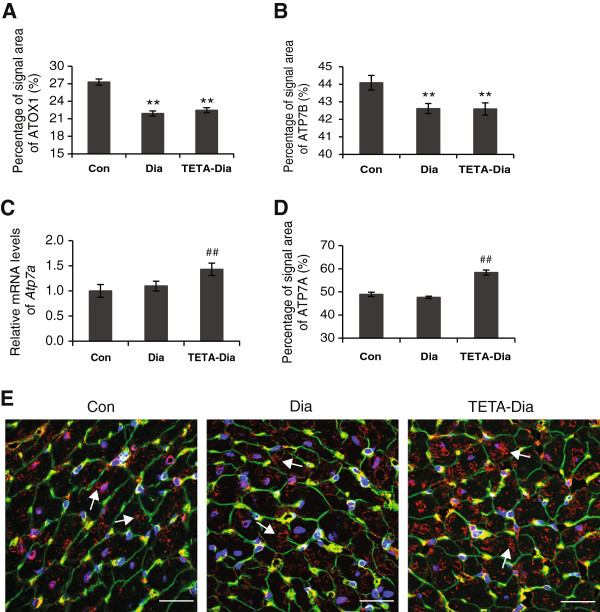
**Expression of analysis of ATOX1**, **ATP7B and ATP7A in LV tissues from control**, **diabetic and TETA**-**treated diabetic rat. A** and **B**: Quantitative analysis of immunofluorescent signal area for protein signals of ATOX1 and ATP7B, respectively. Results were expressed as mean of the percentages of corresponding cross-sectional areas. At least 40 sectional images/group, have been analyzed: **P < 0.01 vs. control. **C**: RT-qPCR analysis for mRNA levels of *Atp7a*. Data are means ± SEM and presented relative to the respective controls, which were set at 1. ^##^*P* < 0.01 vs. diabetic: n = 9/group. **D**: Quantitative analysis of immunofluorescent signal area for ATP7A-protein. Results have been expressed as means of the percentages of corresponding cross-sectional areas. At least 40 sectional images/group, were analyzed: ^##^*P* < 0.01 vs. diabetic. **E**: Representative confocal images (60 ×-objective) of transverse LV-wall sections labeled with anti-ATP7A antibody (*red*). Arrows indicate the peri-nuclear stain and Golgi-network structure: n = 40 sectional images/group: scale bar = 30 μm.

**Figure 8:**
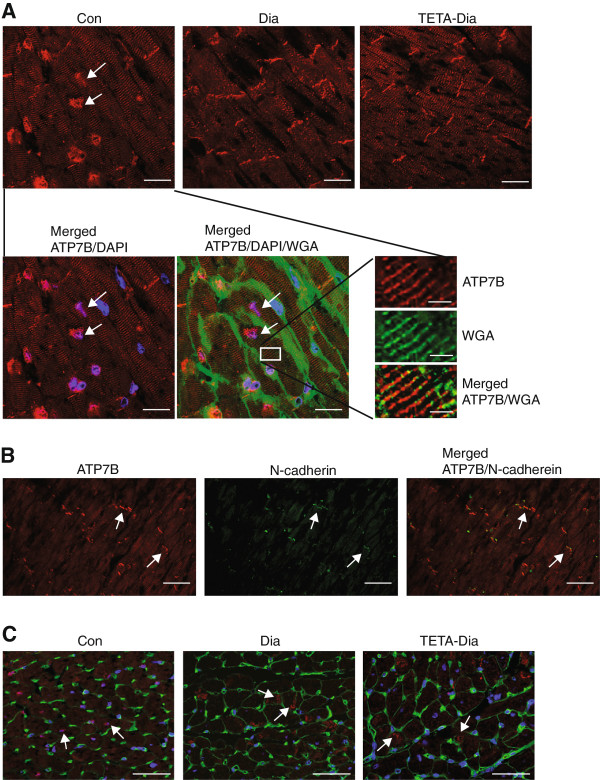
**Immunofluorescent micrographs of ATP7B localization in sections of the LV free**-**wall from control**, **diabetic and TETA**-**treated diabetic rats. A**: Representative confocal images (100 ×-objective) of LV-wall longitudinal sections labeled with anti-ATP7B antibody (*red*). Sections were co-stained for sarcolemma (*green*) and nuclei (*blue*) with WGA-Oregon Green 488 and DAPI, respectively. Arrows indicate the co-staining of nuclei and ATP7B: scale bar = 20 μm. The more highly magnified images show co-localization of ATP7B and T-tubule membranes: scale bar = 4 μm. **B**: Representative confocal images (60 ×-objective) of longitudinal LV-wall sections labeled for ATP7B (*red*) and N-cadherin (*green*). Arrows indicate co-staining at the intercalated disk: scale bar = 30 μm. **C**: Representative confocal images (40 ×-objective) of transverse LV-wall sections labeled with anti-ATP7B antibody (*red*), WGA (*green*), and DAPI (*blue*). Arrows indicate nuclear/peri-nuclear and sarcoplasmic vesicles: scale bar = 50 μm: n = 40 sectional images/group.

Copper supply to ATP7A and ATP7B is mediated by the copper-chaperone protein ATOX1, which binds to and transports copper to compartments containing the target ATPases. In light of their roles in copper regulation, we investigated the myocardial expression of these three proteins. We found no significant changes in the mRNA levels of *Atox1* or *Atp7b* in diabetic or TETA-treated diabetic LV compared to control (data not shown). However, quantitative immunofluorescence of signal areas of ATOX1 and ATP7B, demonstrated that both were decreased in diabetic LV, and remained so following TETA treatment (Figure 7A and 7B).Confocal micrographs of longitudinal LV sections from all treatment groups showed that ATP7B localizes in the nuclear/peri-nuclear region (presumably in the trans-Golgi network), and also with the T-tubules (Figure 8A) and intercalated disks (as indicated by co-staining with N-cadherin) (Figure 8B). Comparative staining of ATP7B in all 3 treatment groups showed that, in control transverse LV sections, ATP7B was once again detected in nuclear and peri-nuclear regions, but this nuclear staining was diminished in diabetic and TETA-treated diabetic LV. Instead, diabetic and TETA-treated diabetic LV had a more striking vesicular localization in the sarcoplasm, which in places was close to the sarcolemmal membrane (Figure 8C). The diminished expression and altered localization of ATP7B, combined with lowered ATOX1 expression, may cause impaired transport of copper to copper-requiring proteins, and indeed efflux from the cell/between cells, providing a putative mechanism for reversing the localized copper imbalances that may occur in diabetic myocardium.By contrast, the mRNA levels and quantitative immunofluorescent signal areas for ATP7A were both unaltered in diabetic LV as compared to control, whereas TETA-treated diabetic LV showed significantly increased ATP7A levels, implying transcriptional and translational up-regulation (Figure 7C and 7D), consistent with TETA-elicited alterations in rates of copper trafficking via ATP7A in the secretory pathway. Confocal micrographs of transverse sections showed that, in control LV, ATP7A was mainly localized to the peri-nuclear region and also showed vesicular staining throughout the sarcoplasm which may represent the intracellular trans-Golgi network membranes (Figure 7E). No notable changes in ATP7A localization in diabetic LV were apparent, whilst TETA-treated diabetic LV showed significantly enhanced vesicular and peri-nuclear staining intensity (correlating to quantitation). These results imply activation of copper translocation by ATP7A in the secretory pathway, which could lead to improve utilization of copper by cuproenzymes in the secretory compartments in response to TETA treatment.

## Discussion

Here we report that a marked deficiency in total copper, of ~50%, occurred in the LV myocardium of diabetic rats with DCM and, strikingly, that there was full restoration of copper to control levels following treatment with the Cu (II)-selective chelator, TETA, [8,48]. We also demonstrate that TETA-mediated restoration of LV copper was accompanied by marked improvement in the structural and functional defects in the LV of rats with DCM, consistent with prior reports [8,48,60]. TETA treatment with the dosage used for the current protocol did not have any adverse effects on cardiac function in non-diabetic control LV, although long-term treatment with higher dosages could be expected to cause symptomatic copper deficiency [62]. We also identified several myocellular copper proteins that respond to TETA treatment in diabetes, but TETA treatment in non-diabetic controls did not alter expression of copper transporters CTR1 and CTR2. Therefore TETA treatment at the dosage employed does not affect copper transport, and further data from TETA-treated non-diabetic controls are not required for interpretation of results of the current study. One question that then arises from these data is that of how copper-chelation successfully ameliorates LV copper deficiency in diabetes?

Physiological copper exists in the body in two valence states, Cu (I) which is mainly localized in the intracellular compartment and comprises ~95% of total body-copper, and Cu (II), which is largely present in the extracellular space and comprises the remaining ~5% [63]. We report here that diabetic rats have lower myocardial copper, a result mainly of decreased intracellular Cu (I), whereas we have shown previously that chelatable extracellular cardiac Cu (II) is increased by ~3-fold in diabetic rats [8]. These results support the hypothesis that the copper-deficient state in the diabetic LV-myocardium is associated with insufficient [Cu (I)]_IC_ but excessive [Cu (II)]_EC_: that is, it reflects an imbalanced distribution of myocardial copper consistent with defective copper uptake into the LV. Heart failure in diabetes may thus be explained, at least in part, by disruption of the distribution of the two copper valence states, and the concomitant myocardial damage that ensues.

Elevated [Cu (II)]_EC_ is thought to promote the increased formation of advanced glycated end-products (AGEs) in collagen, and enhanced TGF-β-evoked collagen deposition [12]. TETA is a selective Cu (II) chelator [45,64] that binds free copper, thus suppressing Cu (II)-catalyzed reactions of reactive oxygen species, such as O_2_^●-^ or H_2_O_2_, to generate HO^●^ radicals in the ECM [55]. Therefore, the improvement in cardiac structure and function caused by TETA treatment may ultimately be evoked, at least in part, by the rebalancing of the intracellular-to-extracellular ratio of the copper valence states. However, little is known of the molecular basis for regulation of relative myocardial Cu (I)/Cu (II) distribution. The molecular mechanisms through which TETA may correct this imbalance were thus a major focus of this study.

Normal cardiac structure and function is reportedly sensitive to marginal copper deficiency: as copper deficiency worsens, the heart is said to become more susceptible to oxidative damage through impairment of its copper-dependent defense mechanisms, such as those catalyzed by SOD1 [25]. Here we have investigated the mechanisms through which impaired cellular copper regulation could alter the biology of proteins in the copper pathways and their potential influence on the LV myocardium. We have provided compelling evidence for dysregulation of cellular copper pathways in the LV in DCM. Key alterations include: (i) decreased expression of the high-affinity copper transporter CTR1; (ii) decreased expression and increased polymerization of the copper-binding/anti-oxidant defense proteins MT1/2; (iii) decreased levels of CCS and SOD1, coupled with diminished SOD1 activity; (iv) decreased expression of ATOX1 and ATP7B, which could well impair copper transport to sites of synthesis of copper-enzymes within the secretory pathway; and (v) translocation of ATP7B, which may alter copper efflux from or between cardiomyocytes.

The diminished expression of myocardial CTR1 protein levels provides a potential mechanism for localized copper deficiency in the diabetic LV. Unaltered *Ctr1* mRNA levels are consistent with the idea that this copper deficiency may well be generated at a translational rather than transcriptional level. The findings of deficient total copper (mainly Cu (I)) in the LV coupled with substantively diminished CTR1 at the cell membrane, and with increased internalisation and lowered overall levels of the transporter, are consistent with a mechanism known to occur in several mammalian cell types, namely copper-evoked endocytosis [65,66] and degradation [65]: these findings are consistent with the presence of elevated Cu (II) in the ECM of the coronary vasculature in the diabetic heart, which could well trigger this defect [8] and thus cause heart failure in diabetes [16]. The disorganised pattern of CTR1 distribution in the T-tubular region of the diabetic LV is also noteworthy. Consistently, it has been reported that ventricular myocytes from diabetic animals with heart failure possess a sparse, irregular T-tubule system [67]: it is possible that this disorganization could contribute to impaired myocardial copper uptake. Furthermore, the T-tubules are an important determinant of cardiomyocyte function, especially as they are the main site of excitation-contraction coupling [10]. Therefore the observed structural changes at this subcellular site of CTR1 localization may point to a link between defective myocardial copper uptake and impaired myocardial contractility [9,10], implying a potential role of myocellular copper homeostasis in the regulation of excitation-contraction coupling: however, myocellular copper currently has no known role in myocardial excitation-contraction coupling [39]. By contrast, since TETA treatment did not correct diminished CTR1 levels in diabetes, CTR1 is unlikely to play a role in the TETA-evoked correction of LV copper levels and function.

Contrastingly, TETA treatment increased the expression of *Ctr2* mRNA and protein compared to untreated diabetic values but not to untreated control values, suggesting the effects of TETA on these pathways occurred only in diabetes. TETA treatment also enhanced CTR2 localization at the cell periphery, specifically at the outer sarcolemmal membrane and the intercalated disk region, where it could increase copper uptake from the extracellular space and neighboring cardiomyocytes, respectively. Therefore, elevation of CTR2 is a candidate mechanism whereby TETA can restore copper levels in diabetic LV. There is evidence that cells possess more than one copper uptake pathway. Thus, dietary copper supplementation of pregnant mice did not rescue *Ctr1*^−/−^ offspring, suggesting that *Ctr1*^−/−^ embryos cannot acquire copper because of the lack of the plasma membrane CTR1 transporter; however, CTR1-deficient mouse embryonic cells possess a second, CTR1-independent copper transport system [68]. CTR2 localizes in part to the cell membrane, and cells lacking CTR2 have lower copper accumulation [69]. Therefore, although CTR2 is a lower-affinity copper transporter than CTR1, TETA may nevertheless correct LV-copper levels by up-regulating CTR2, thereby increasing copper import. Moreover, TETA-evoked increase in the sarcolemmal localization of CTR2 contrasts with the observed enhancement of CTR2 localization in the vesicular compartments in diabetic LV: a vesicular localization for CTR2 has previously been reported, where it was noted to co-localize with both lysosomes and late endosomes [70]. Diabetes-mediated elevations in CTR2 expression in vesicular membranes are possibly the endogenous compensatory response by which copper released from copper proteins by lysosomal degradation is recycled into the cytosol and thus made available for cellular utilization in response to lowered copper uptake by CTR1: this could happen without changing total cellular copper levels. Moreover, the elevated recycling of copper into the cytosol via CTR2 could also serve as a signal of increasing intracellular copper levels, in turn further lowering copper uptake by CTR1. Thus CTR2 and CTR1 show opposing changes in expression and thus, quite possibly, opposing roles in diabetes-induced LV-copper deficiency. Lastly, our findings in diabetic rats contrast with the lowered expression of CTR2 in the hearts of rats with diet-induced systemic copper-deficiency [71]: this contrast points to a distinct pathogenic mechanism in the regulation of copper uptake in the LV myocardium of the diabetic rat in the context of the overall systemic copper overload that occurs in diabetes [8,45].

Metallothioneins are one of the major classes of copper-binding proteins contributing to the regulation of intracellular copper homeostasis and protection against excess cytoplasmic copper [61,72], acting through their actions as a scavenger of transition metal atoms and radicals [72]. Previous studies have implied that oxidative stress induced by chronic hyperglycemia can impair intracellular copper homeostasis in the diabetic heart, in part by suppressing myocardial MT expression [61,73]. Here, we detected decreased expression but increased polymerization of MT in diabetic myocardium which may lead to decreased availability of redox-responsive forms of MT, and thus to decreased protection against copper-mediated toxicity. The lowering of total MT will lead to a concomitant reduction in cytoplasmic copper-binding capacity, which is consistent with the lowered intracellular copper levels we observed, most likely attributable to reduced CTR1-mediated copper influx. The decreased expression of the highly polymerized, ~70-kD isoform of MT present after TETA treatment is consistent with the decrease in levels of copper-containing MT associated with decreased cellular oxidation. This finding correlates with improved functional activities of MT, and is consistent with a process of MT-mediated correction in myocardial cytoplasmic copper levels.

Here we also found evidence of decreased copper supply to SOD1 via CCS in diabetic myocardium, which could lead to the measured deficiencies in SOD1 activity. The catalytic function of SOD1 is dependent on copper redox chemistry at its active site, and is thus potentially regulated by rates of cellular copper supply: the observed relative inactivity of SOD1 in diabetic myocardium is consistent with deficient copper metalation of apo-SOD1. It has been reported that SOD1 with lowered copper content is less catalytically active, and may also be unstable and degraded faster than the normally-metalated enzyme [74]. The decreased activity of SOD1 could lead to diminished anti-oxidant protection thus enabling enhanced oxidative damage in the diabetic LV-myocardium. In contrast to other reports showing up-regulation of CCS protein caused by dietary copper-deficiency in rats [75], our study demonstrates lowered levels of CCS in copper-deficient LV myocardium, consistent with a contrasting role of CCS in response to the changes of copper status under diabetic conditions. TETA treatment rectified CCS levels and the activity of SOD1, consistent with restoration of copper-supply to SOD1 via CCS, reversing deficient SOD1 activity, and contributing to demonstrated restoration of myocardial anti-oxidant defenses [60].

ATP7A and ATP7B contribute to the maintenance of copper-dependent enzyme activity by delivering copper to the lumen of the secretory pathway in the trans-Golgi network, where copper metalation generates active cuproenzymes. These ATPases can also contribute to the maintenance of intracellular copper concentrations by transporting copper into secretory vesicles from the trans-Golgi network, which shuttle to the plasma membrane for copper excretion [76]. Here we found that, in diabetic LV, protein levels of both ATOX1 and ATP7B were decreased, consistent with possible impairment of copper supply for activation of cuproenzymes. The decreased localization of ATP7B to the sarcolemmal membrane and intercalated disc regions in diabetes could limit intercellular transport of copper. Cumulatively these mechanisms could contribute to the pattern of copper deficiency and impaired cardiac function [9,10]. We also found that the ATP7A copper transporter, which is thought to function mainly in intestinal copper acquisition [39], is expressed in the myocardium. TETA enhanced the expression of ATP7A mRNA and protein, which could compensate and help to rectify impaired copper delivery to the secretory pathway/intercellular transport by defective ATP7B action. These effects could contribute to the restoration of physiological copper homeostasis and biosynthesis of active copper-dependent enzymes in the myocardium, in parallel to the TETA-mediated restoration of the CCS-SOD1 pathway. The observed up-regulation of ATP7A with enhanced peri-nuclear localization following TETA treatment is consistent with enhanced delivery of copper to the trans-Golgi network, presumably aiding copper supply for newly-synthesized copper proteins.

Some of the observed abnormalities, for example lowered levels of CTR1, CCS and SOD1, probably contribute to the causation of the localized copper-deficiency state in diabetic myocardium or to its adverse consequences, and thus to the functional impairment observed in the hearts of diabetic animals [8,45,48]; other effects, such as elevated CTR2 and lowered MT1/2 and ATP7B, may reflect endogenous responses directed towards ameliorating copper deficiency or its impacts.

How might TETA exert its intracellular effects? We have previously shown that TETA selectively binds excess Cu (II) in diabetic individuals and elicits its removal from the body via urinary excretion, through studies where the following methods were applied: electron paramagnetic resonance spectroscopy; X-ray crystallography; potentiometric, spectrophotometric and mass-spectrometric analysis of complex formation between Cu (II), and TETA and its metabolites; and clinical studies [8,45,55,64]. Thus TETA can remove excess Cu (II) from the ECM, probably by binding and removing it from pathogenic binding sites such as those in AGE-modified collagen [77,78]. AGE-coordinated Cu (II) almost certainly remains catalytically active, and could therefore bind to the external, high-affinity Cu (II)-binding site present near the NH_2_-terminus of CTR1 [79]. Thus, elevated Cu (II) bound to AGE-modified collagen in diabetic individuals could participate in the modulation of cell copper metabolism through binding to CTR1, perhaps resulting in its translocation away from the cell membrane as shown herein. However, TETA is also known to cross cell membranes, probably via mechanisms employed by its physiological homologues, spermine and spermidine [80]. For example, there is substantive evidence that TETA can traverse cell membranes in the gut and kidney via a Na^+^/spermine-antiporter-mediated mechanism [81]. It thus has the potential to exert direct effects in the intracellular compartment.

TETA forms two main metabolites in the body, monoacetyl-TETA and diacetyl-TETA [54,82-85]. Both acetyl metabolites are strong chelators in their own right, although their affinities for Cu (II) are substantially less than that of the parent compound [64]. There is evidence that monoacetyl-TETA may contribute to the overall response to treatment in diabetic patients [84]. There are no published reports known to us, however, describing the uptake of TETA directly into cardiomyocytes, so whether TETA and its metabolites could act directly within the intracellular compartment to influence copper homeostasis remains to be determined.

These studies have also provided a new molecular explanation linking the therapeutic effects of TETA in diabetes to the restoration of myocardial copper content. We provide new evidence of intracellular targets of TETA treatment, whose amended expression/activity restore antioxidant defenses, which include: (i) decreasing the polymerization of MT1/2, consistent with diminished pro-oxidant stress; (ii) restoring LV-myocardial copper uptake, at least in part via increased expression of CTR2 in the sarcolemma; (iii) increasing copper supply to SOD1 via increased expression of CCS, leading to restoration of SOD1 function; (iv) increasing expression and localization of ATP7A in the trans-Golgi region, by which it could improve copper translocation into the lumen of the secretory pathway for synthesis of active cuproenzymes. Our results indicate that hyperglycemia-induced cell-copper imbalance in cardiomyocytes might be rectified by TETA treatment via increased sarcolemmal copper importation coupled with compensatory modifications in the export pathway. Cu (II)-chelation with TETA treatment could thus allow the (partial) restoration of copper delivery to intracellular sites of utilization and storage.

## Conclusions

In conclusion, we have identified for the first time, a diabetes-induced process that impairs myocellular copper-transport, leading to severe localized copper deficiency in the LV-myocardium. This myocardial copper deficiency elicits severe LV dysfunction that leads to DCM, which is reversed by TETA treatment in the model we employed here. Our parallel investigations in patients with LV hypertrophy and diastolic dysfunction caused by type-2 diabetes, have indicated that a similar process probably occurs in humans with DCM, who show signs of systemic copper overload [45], and in whom excessive LV mass was markedly improved by TETA treatment [8,48].We also show here that this newly-recognized pathway can serve as a target for drug discovery. For example, TETA treatment normalized myocardial copper content and could thus contribute to the maintenance or restoration of normal myocardial function in diabetes, by restoring myocellular copper transport and antioxidant defenses. Our findings provide a worked example for targeting a newly-recognized myocardial copper transport pathway for pharmaceutical discovery, using an approach based on highly-selective Cu (II) chelation, which is shown here to restore several defective, myocardial copper-trafficking processes that would otherwise conspire to cause DCM and heart failure. These findings have the potential to alter the management of DCM.

## Abbreviations

ATOX1: Antioxidant protein 1; ATP7A: copper-transporting-ATPase-1 (Menkes’ disease protein); ATP7B: copper-transporting-ATPase-2 (Wilson’s disease protein); CCS: Copper chaperone for superoxide dismutase; CTR1: Copper transporter 1; CTR2: Copper transporter 2; Cu (I): Univalent copper; Cu (II): Divalent copper; DCM: Diabetic cardiomyopathy; ECM: Extra-cellular matrix; IC: Intracellular; LV: Left-ventricular; MT1: Metallothionein 1; MT2: Metallothionein 2; O_2_^●-^: Superoxide anion; PIXE: Particle-induced x-ray emission spectroscopy; STZ: Streptozotocin; SOD1: Superoxide dismutase 1; SOD3: Superoxide dismutase 3; TETA: Triethylenetetramine.

## Competing interests

GJSC is named as an inventor on patents that disclose the treatment of diabetes with TETA but declares no other related competing interests. All the other authors have declared that no competing interests exist with respect to this work.

## Authors’ contributions

SZ conceived and designed the study, wrote the manuscript and was responsible for acquisition, interpretation and analysis of data. HL participated in experimental design, acquisition and analysis of data. GVA, CCHC, SH, UN, JK, BB and YSC contributed to acquisition and analysis of data. LZ contributed to data analysis and revision of manuscript. SM contributed to revision of manuscript. JX and DG acquired data. ARJP contributed to experimental design and revision of manuscript. GJSC was responsible for conception and design of the study, interpretation of data, wrote the manuscript, and bears overall responsibility for the study and manuscript. All authors read and approved the final manuscript.

## Supplementary Material

Additional file 1 Evaluation of robust normalizers suitable for qPCR analysis for mRNA levels in cardiac tissues from non-diabetic, STZ-diabetic and TETA-treated diabetic rats. Click here for file
