# Supplementary material for: Diabetic cardiomyopathy is associated with defective myocellular copper regulation and both defects are rectified by divalent copper chelation
Source: Cardiovasc Diabetol. 2014 Jun 14;13:100. doi: 10.1186/1475-2840-13-100 (PMC4070334; doi:10.1186/1475-2840-13-100)
Supplement: Additional file 1 — Evaluation of robust normalizers suitable for qPCR analysis for mRNA levels in cardiac tissues from non-diabetic, STZ-diabetic and TETA-treated diabetic rats. [file 1475-2840-13-100-S1.docx]

**Supplemental Material**

**Diabetic cardiomyopathy is associated with defective myocellular copper regulation and both defects are rectified by divalent copper chelation**

**Details of Methods**

**Ethical and regulatory considerations**

All experimental protocols were approved by the Animal Ethics Committee of the University of Auckland. The study was performed according to the ‘Guide for the Care and Use of Laboratory Animals’ [[1](#_ENREF_1)], and this manuscript is consistent with the ‘ARRIVE guidelines for the reporting of animal research’ [[2](#_ENREF_2)].

**Animal studies**

Protocols for the induction of diabetes in rats, and TETA treatment were as previously described [[3](#_ENREF_3), [4](#_ENREF_4)]. Diabetes was induced by a single intravenous injection of STZ (Streptozotocin, 55 mg/kg body-weight; Sigma) into the tail-veins of adult male Wistar rats (6-7 weeks of age; 220-250 g). Control animals received a saline injection instead of STZ. TETA was administered via the drinking water (20 mg/day per rat, Fluka), beginning at 8 weeks after STZ injection. LV tissues from each treatment group (non-diabetic- control, diabetic, and TETA treated-diabetic) were collected after 8-weeks’ treatment. TETA-treated controls were not included in this study since we have previously shown that they are not required for interpretation of results [[3](#_ENREF_3), [4](#_ENREF_4)].

**Choice of TETA dosage**

The dosage used here was based on those employed in known clinical applications of TETA (TETA dihydrochloride or trientine) in the treatment of patients with Wilson’s disease, and for the experimental therapy of diabetes, as described [[5](#_ENREF_5)]. In brief, dosages employed for the treatment of Wilson’s disease in adults typically vary from 750–2000 mg/day (equivalent to ~11-29 mg/kg-day in 70-kg adults) [[6](#_ENREF_6)]. Here, we administered TETA dihydrochloride in the drinking water to diabetic rats at ~17 mg/day (equivalent to ~68 mg/kg-day of trientine in 250-g rats). This dosage is supported by our published dose-rising phase-1 clinical trial, where we showed that dosages of 1200 and 3600 mg/day (equivalent to ~17 and 51 mg/kg-day in 70-kg adults) were effective and well tolerated in healthy adult human volunteers [[7](#_ENREF_7)], and also by our phase-2 trial where 1200 mg/day of trientine (~17 mg/kg-day in 70-kg adults) administered for 12 months markedly improved LV mass in T2D patients with LV hypertrophy [[8](#_ENREF_8)].

**Choice and validation of animal model**

We have extensively validated the STZ-based model we chose to employ here by showing that these diabetic rats respond both to untreated diabetes and following TETA treatment, in ways that closely reflect responses in patients with T2D [[3-5](#_ENREF_3), [8-11](#_ENREF_8)]. To date, we have shown that TETA treatment exerts substantial effects to restore LV mass in patients with LV hypertrophy [[8](#_ENREF_8)], similar to its effects in rats [[3](#_ENREF_3), [4](#_ENREF_4)], and that the dosage-responsiveness of TETA-mediated removal of copper from the body is similar in rats [[3](#_ENREF_3)] and human volunteers [[7](#_ENREF_7), [12](#_ENREF_12)]. These findings provide strong support for our use of this model.

**Measurement of ex-vivo cardiac function**

We measured cardiac function in isolated, *ex-vivo* perfused working hearts, as previously detailed [[3](#_ENREF_3), [4](#_ENREF_4)]. On the experimental day, rats were anesthetized (isoflurane), heparinized (1,000 IU/kg i.v.), and hearts excised and immersed in 4°C Krebs-Henseleit bicarbonate buffer (KHB). Retrograde (Langendorff) perfusion was established (KHB, 37°C, gassed with O_2_:CO_2_ 95:5 (vol/vol)). Working-mode perfusion was then established (preload, 10 cmH_2_O; afterload, 55.9 mmHg) with pacing (300 bpm; Digitimer). Intrachamber LV pressure (SP855; AD Instruments), aortic pressure (PX23XL, Stratham Gould), and aortic (Transonic T206) and coronary flows were measured; pressure and flow data were recorded (Powerlab16s, ADI); and maximal rates of ventricular pressure development (+dP_LV_/dt) and relaxation (-dP_LV_/dt) were derived. Atrial filling pressure was decreased (5 cmH_2_O) and then increased (seven equal steps of 2.5 cmH_2_O to 20 cmH_2_O [final]), and 1-min averages were extracted. Filling pressure was then fixed at 10 cmH_2_O, and afterload at 75 mmHg.

**Measurement of tissue copper content**

Copper concentrations were determined in dry *ex vivo* LV-tissue by using a reference method: particle-induced x-ray emission spectroscopy (PIXE) coupled with Rutherford backscattering spectrometry (RBS) [[13](#_ENREF_13)]. The calibration, measurements, and limits of detection were based on the areas of the K_α_ x-ray peaks as measured by the software package GUPIX Elemental, and concentrations were extracted from PIXE spectra using GUPIX software (University of Guelph, ON, Canada), as previously described [[5](#_ENREF_5), [14](#_ENREF_14)].

**Measurement of mRNA by RT-qPCR**

Here, reverse-transcriptase quantitative polymerase chain reactions were performed according to ‘The Minimum Information for Publication of Quantitative Real-Time PCR Experiments (MIQE)’ guidelines [[15](#_ENREF_15)]. Total RNA was extracted from rat LV tissues that had previously been preserved (RNAlater, Ambion), by using an RNeasy mini kit (Qiagen), and was reverse-transcribed into cDNA using a first-strand cDNA synthetase kit (Roche Diagnostics). Real-time qPCR protocols were designed based on the MIQE guidelines and performed using the SYBR Green technology on a LightCycler rapid thermal cycler (Roche Diagnostics), in accordance with the manufacturer’s instructions. Quantification results for mRNA levels for each target gene were normalized to the geometric mean of the expression of three optimal reference genes for the study of gene expression in cardiac tissue (*Rpl13a, Tbp* and *Ndc1*). PCR primers employed are as listed in Supplementary Table 1.

**Western Blot Analysis**

Protein extracts from LV tissues were prepared by adding ice-cold RIPA buffer supplemented with a protease inhibitor cocktail (Roche, Basle, Switzerland), and homogenised using a TissueLyser II (Qiagen). After centrifugation (20 min, 16,500 x *g*, 4°C), protein concentrations of supernatants were determined by BCA assay (Thermo Scientific, Auckland, New Zealand). Western blotting was performed as previously described [[16](#_ENREF_16)]. Briefly, 25 µg of each sample was loaded onto a 12% Bis-Tris-polyacrylamide gel (Invitrogen) and transferred to nitrocellulose membranes.

Blots were first probed with specific antibodies as follows: CTR1 (sc-66847, Santa Cruz Biotechnology Inc); CTR2 (sc-104852, Santa Cruz Biotechnology Inc); MT1/2 (sc-11377, Santa Cruz Biotechnology Inc), and then incubated with an HRP-conjugated IgG, (dilution 1:10,000, Jackson Laboratories). Specific protein bands were detected by using an enhanced chemiluminescent substrate (ECL Plus, GE Healthcare, Buckinghamshire, UK) and imaged using an LAS 3000 image reader (Fuji Photo Film Co. Ltd, Tokyo, Japan). Ponceau-S staining was used as the reference for loading controls, and band intensities were measured using MultiGauge software v2.0 (Fuji Photo Film).

**Cu/Zn-SOD1 ELISA**

LV tissues were weighed and minced into small pieces before homogenization in phosphate-buffered saline (pH 7.2; 20 µl/mg tissue) using a TissueLyser II (Qiagen). The resulting suspension was subjected to two freeze-thaw cycles and then centrifuged (5 min, 5000×*g*, 4°C). Thereafter, supernatants were removed and total-protein concentrations determined by BCA assay (Thermo Scientific). SOD1 concentrations were measured using an ELISA kit according to the manufacturer’s protocol (USCN Life Science; Wuhan, Hubei, PRC). Data were detected by spectrophotometry (450 nm, microplate reader; SpectraMAX 340, Molecular Devices; Sunnyvale, CA) and the concentration of SOD1 (ng/mL) in the sample supernatants calculated by comparing the O.D. of the samples to a standard curve. Results were then converted to ng/mg total protein and expressed as concentrations relative to the corresponding control group.

**Cu/Zn-SOD1 activity assay**

LV tissues were homogenized in ice-cold 0.1M Tris/HCl, pH 7.4 containing 0.5 % Triton X-100, 5 mM ME and 0.1 mg/ml PMSF using a TissueLyser II (Qiagen). The crude tissue homogenates were then centrifuged (14,000x*g*, 5 min, 4°C) and the supernatants collected: these contain total SOD activity from combined cytosolic and mitochondrial sources. Following incubation with 0.4 vol of a solution containing ethanol and chloroform (25:15 vol/vol) to inactivate the mitochondrial SOD2 [[17](#_ENREF_17)], mixtures were centrifuged at 5000 x g for 15 min and aliquots of the supernatant used to measure protein concentration by BCA assay (Thermo Scientific), and Cu/Zn-SOD activity using the Superoxide Dismutase Activity Assay kit (Abcam). The latter works by measuring the inhibition of the reduction of a water-soluble tetrazolium salt (WST-1) which produces a water-soluble formazan dye upon reduction by an O_2_^●-^ radical, which are generated by xanthine oxidase and inhibited by SOD. One unit of SOD activity is the amount necessary to inhibit the xanthine oxidation by 50%. The IC_50_ (50% inhibition activity of SOD) was detected by a colorimetric method at 450 nm using an absorbance microplate reader (SpectraMAX 340, Molecular Devices) and the results expressed as per mg of total protein.

**Immunofluorescence analysis of LV free-wall tissue**

Immunofluorescent staining was performed using standard techniques as described. Briefly, 6 µm LV sections frozen in OCT were cut, fixed in acetone, and blocked with 5% (vol/vol) normal donkey serum (NDS) in PBS. They were then incubated overnight at 4^o^C with primary antibody in 5% NDS (vol/vol). Primary antibodies used in these experiments were: anti-CTR1 (at 1:200 vol/vol final dilution, Abnova); anti-CTR2 (1:50, Santa Cruz); MT1/2 (1:100, Abcam); CCS (1:200, Novus Biologicals); ATOX1 (1:100, Santa Cruz Biotechnology Inc); ATP7A (1:250, Abcam), ATP7B (1:150, Novus Biologicals), and N-cadherin (1:50, Abcam). Following 3 x washes with PBS, sections were incubated with appropriate fluorescently-labeled (-FITC or -Rhodamine Red, as required) donkey anti-rabbit/mouse/chicken IgG secondary antibodies (Jackson ImmunoResearch). After incubation, sections were washed and incubated overnight with WGA-Oregon Green 488 (1:5000; Molecular Probes) in PBS at 4^o^C. Finally, sections were washed again and mounted with Prolong Gold anti-fade reagent with DAPI (Invitrogen). The immunofluorescent images were captured at 40X, 60X or 100X magnifications using a confocal fluorescent microscope (Olympus FV1000, Fuji).

The fluorescently-labeled signals from each image were processed and quantified using a custom-written IDL analysis program based on signal-thresholding, as previously described [[5](#_ENREF_5), [16](#_ENREF_16)]. Briefly, the original image was masked to exclude non-labeled areas. The threshold for quantifying fluorescent labeling was then established, based on information in the masked image’s histogram compared with the original image. The criteria for the thresholding process were maintained equivalent across all experimental groups. Results were expressed as percentages of corresponding total cross-sectional areas. To avoid any possible confounding effect of orientation and origin of the muscle fibers on the signal quantification, at least five transverse optical-sectional images from each section of the LV-myocardium, two sections from each animal, and four animals per group, were analyzed.

**Statistical analysis**

Data have been expressed as mean ± SEM unless stated otherwise. Significance of between-group differences has been determined by unpaired Student’s *t-*tests, one-way ANOVA, or two-way ANOVA, as appropriate. The primary *a priori* null hypothesis in each contrast was that there was no difference between signals from TETA-treated diabetic and untreated diabetic states. A second between-group comparison was performed in each case, with the *a priori* null hypothesis being that there was no difference between signals from control and diabetic animals: this latter contrast was included to ensure that effects of diabetes were present as a starting condition for comparison with TETA-treated samples. Where stated in the figure legends, correction for multiple-comparisons has been applied. All contrasts were two-tailed, and values of *P <* 0.05 have been considered significant.

**Supporting information**

**Selection of robust normalizers suitable for qPCR analysis for mRNA levels in cardiac tissues from non-diabetic, STZ-diabetic and TETA-treated diabetic rats**

Gene expression analysis by real-time qPCR is a powerful method for analysing changes in gene expression in various biological tissues[[18](#_ENREF_18), [19](#_ENREF_19)]. Data normalization using sufficiently robust normalizers is crucial for accurate quantification of mRNA expression [[19-22](#_ENREF_19)]. In this experiment, we measured expression-stability of 12 candidate reference genes (CRGs) (*18S rRNA, Gapdh, Actb, B2m, Hprt1, Ubc, Ndc1, Ppia, Rpl13a, U2af, Tbp and Ywhaz*) with the aim of identifying stably-expressed CRGs that are suitable for use as robust normalizers for qPCR analysis of mRNA levels in cardiac tissues from non-diabetic, STZ-diabetic and TETA-treated diabetic rats. The objective of selecting robust normalizers was to avoid errors that all too frequently occur with the use of single gene normalizers. This approach is consistent with international best-practice recommendations for quantitative measurements of mRNAs by RT-qPCR [[15](#_ENREF_15)].

## RT-qPCR was performed using a LightCycler 480 System (Roche) according to the manufacturer’s instructions, with SYBR Green I Master Mix (Roche). Reactions were performed in triplicate in 384-multiwell plates with pre-incubation for 5 min at 95°C, followed by 45 cycles, each of 10 s at 95°C, 15 s at 60°C and 20 s at 72°C. In each qPCR run, a standard curve for each respective gene was generated using the 6-point dilution series (which ranged between 0.01 and 25 ng). To check for possible genomic DNA contamination and multiplex products, a melting curve was generated for every run. The sequences of each gene-specific primer pair used are shown in Table 1.

## For data analysis, Threshold-values (crossing point Cp) and relative mRNA concentrations of each given reference gene were obtained for every qPCR reaction using LC 480 Software v1.5 (Roche). Stability of each reference genes was analyzed using the geNorm (GN) [[20](#_ENREF_20)] and NormFinder (NF) (2, 4) algorithmic approaches: <http://medgen.ugent.be/~jvdesomp/genorm/> and <http://www.mdl.dk/publicationsnormfinder.htm>, respectively. We found that *Rpl13a, Tbp* and *Ndc1* are most stably-expressed CRGs, and the geometric mean of the expression of these three CRGs forms a best robust normalizer for qPCR analysis in the selected cardiac tissues. This study has therefore used this robust normalizer to measure relative mRNA expression of genes involved in regulation of copper transport, as presented in Table 1 below. *S*tatistical analysis was performed using unpaired Student’s *t*-tests for pairwise comparisons and/or ANOVA for between-group comparisons. This statistical approach was deemed adequate as the comparisons made were *a priori* defined.

**Supplemental Table I. Sequences of primers used for qPCR analysis.**

| **Genes** | **Symbol** | **Sequences (5’ – 3’)** |
| --- | --- | --- |
| 18S ribosomal RNA | *18S* | aaatcagttatggttcctttggtc  gctctagaattaccacagttatccaa |
| beta-actin | *Actb* | gctgtattcccctccatcgtg  cacggttggcctttagggttcag |
| Glyceraldehydes-3-phosphate dehydrogenase | *Gapdh* | tgacgtgccgcctggagaaa  agtgtagcccaagatgcccttcag |
| Beta-2-microglobulin | *B2m* | ttctggtgccttgtctcactga  cagtatgttcggcttcccattc |
| Hypoxanthine guanine phosphoribosyl transferase | *Hprt1* | gcttgctggtgaaaaggacctctcgaag  ccctgaagtactcattatagtcaagggcat |
| Ubiquitin C | *Ubc* | aggtcaaacaggaagacagacgta  tcacacccaagaacaagcaca |
| Nucleoporin 1 | *Ndc1* | ttcccaaagcatggattagc  cagccagacgtggtagagta |
| Peptidylprolyl isomerase A | *Ppia* | cgcgtctccttcgagctgtttg  tgtaaagtcaccaccctggca |
| Ribosomal protein L13a | *Rpl13a* | acaagaaaaagcggatggtg  ttctcctccagagtggctgt |
| U2 auxiliary factor 36 kDa subunit | *U2af* | ccattgccctcttgaacatt  cttccccgtacttctcttcc |
| Phospholipase A2 | *YWHAZ* | tcttgtcaccaaccattcca  aggggaagcgggtatcttag |
| TATA box binding protein | *Tbp* | agaacaatccagactagcagca  gggaacttcacatcacagctc |
| Copper uptake transporter 1 | *Ctr1* | caacacacctggagaaatgg  cgggctatcttgagtccttc |
| Copper uptake transporter 2 | *Ctr2* | tgtaactttgaggccagagaga  gcccatgaggtacttgaacg |
| Copper chaperone for superoxide dismutase 1 | *Ccs* | ctgtgcacaagaccctgaaa  ccatctggttctccaactgaa |
| Superoxide dismutase 1 | *Sod1* | ggtccacgagaaacaagatga  caatcacaccacaagccaag |
| Antioxidant 1 copper chaperone | *Atox1* | aaagcggtctcctaccttgg  agctggactgagcagttggt |
| Copper-transporting ATPase, alpha polypeptide | *Atp7a* | agcttcaggaggagggaaag  agctgcttcgatggctacat |
| Copper-transporting ATPase, beta polypeptide | *Atp7b* | aacggcgtcctaatcaagg  tgcccgttttgtcaaacata |
| Metallothionein 1 | *Mt1* | agtgacgaacagtgctgctg  tcggtagaaaacggggttta |
| Metallothionein 2 | *Mt2* | gcgatctctcgttgatctcc  gcatttgcattgtttgcatt |

**Supplemental References**:

1. Committee for the Update of the Guide for the Care and Use of Laboratory Animals: **Guide for the Care and Use of Laboratory Animals**, Eighth edn. Washington, D.C.: Institute for Laboratory Animal Research, Division on Earth and Life Studies, National Research Council of the National Academies, The National Academies Press; 2011.

2. Kilkenny C, Browne WJ, Cuthill IC, Emerson M, Altman DG: **Improving bioscience research reporting: the ARRIVE guidelines for reporting animal research**. *PLoS Biol* 2010, **8(6)**:e1000412.

3. Cooper GJS, Phillips ARJ, Choong SY, Leonard BL, Crossman DJ, Brunton DH, Saafi L, Dissanayake AM, Cowan BR, Young AA *et al*: **Regeneration of the heart in diabetes by selective copper chelation**. *Diabetes* 2004, **53**(9):2501-2508.

4. Lu J, Pontré B, Pickup S, Choong SY, Li M, Xu H, Gamble GD, Phillips ARJ, Cowan BR, Young AA *et al*: **Treatment with a copper-selective chelator causes substantive improvement in cardiac function of diabetic rats with left-ventricular impairment**. *Cardiovasc Diabetol* 2013, **12**:28.

5. Zhang L, Ward M-L, Phillips ARJ, Zhang S, Cannell MB, Cooper GJS: **Protection of the heart by treatment with a copper(II)-selective chelator reveals a novel mechanism underlying diabetic cardiomyopathy**. *Cardiovasc Diabetol* 2013, **12**:123.

6. Dahlman T, Hartvig P, Löfholm M, Nordlinder H, Lööf L, Westermark K: **Long-term treatment of Wilson’s disease with triethylene tetramine dihydrochloride (trientine)**. *Q J Med* 1995, **88**:609-616.

7. Cho HY, Blum RA, Sunderland T, Cooper GJS, Jusko WJ: **Pharmacokinetic and pharmacodynamic modeling of a copper-selective chelator (TETA) in healthy adults**. *J Clin Pharmacol* 2009, **49**(8):916-928.

8. Cooper GJS, Young AA, Gamble GD, Occleshaw CJ, Dissanayake AM, Cowan BR, Brunton DH, Baker JR, Phillips AR, Frampton CM *et al*: **A copper(II)-selective chelator ameliorates left-ventricular hypertrophy in type 2 diabetic patients: a randomised placebo-controlled study**. *Diabetologia* 2009, **52**(4):715-722.

9. Cooper GJS: **Therapeutic potential of copper chelation with triethylenetetramine in managing diabetes and Alzheimer’s disease**. *Drugs* 2011, **71**:1281-1320.

10. Cooper GJS: **Selective divalent copper chelation for the treatment of diabetes mellitus**. *Curr Med Chem* 2012, **19**:2828-2860.

11. Cooper GJS, Chan YK, Dissanayake AM, Leahy FE, Keogh GF, Frampton CM, Gamble GD, Brunton DH, Baker JR, Poppitt SD: **Demonstration of a hyperglycemia-driven pathogenic abnormality of copper homeostasis in diabetes and its reversibility by selective chelation: quantitative comparisons between the biology of copper and eight other nutritionally essential elements in normal and diabetic individuals**. *Diabetes* 2005, **54**(5):1468-1476.

12. Lu J, Poppitt SD, Othman AA, Sunderland T, Ruggiero K, Willet MS, Diamond LE, Garcia WD, Roesch BG, Cooper GJS: **Pharmacokinetics, pharmacodynamics, and metabolism of triethylenetetramine in healthy human participants: an open-label trial**. *J Clin Pharmacol* 2010, **50**(6):647-658.

13. Bertrand M, Weber G, Schoefs B: **Metal determination and quantification in biological materials using particle induced X-ray emission**. *Trends Anal Chem* 2003, **22**:254-262.

14. Gong D, Lu J, Chen X, Reddy S, Crossman DJ, Glyn-Jones S, Choong YS, Kennedy J, Barry B, Zhang S *et al*: **A copper(II)-selective chelator ameliorates diabetes-evoked renal fibrosis and albuminuria, and suppresses pathogenic TGF-beta activation in the kidneys of rats used as a model of diabetes**. *Diabetologia* 2008, **51**(9):1741-1751.

15. Bustin SA, Benes V, Garson JA, Hellemans J, Huggett J, Kubista M, Mueller R, Nolan T, Pfaffl MW, Shipley GL *et al*: **The MIQE guidelines: minimum information for publication of quantitative real-time PCR experiments**. *Clin Chem* 2009, **55**(4):611-622.

16. Zhang L, Cannell MB, Phillips AR, Cooper GJS, Ward ML: **Altered calcium homeostasis does not explain the contractile deficit of diabetic cardiomyopathy**. *Diabetes* 2008, **57**(8):2158-2166.

17. Weisiger RA, Fridovich I: **Superoxide dismutase: organelle specificity**. *J Biol Chem* 1973, **248**:3582-3592.

18. Van Guilder HD, Vrana KE, Freeman WM: **Twenty-five years of quantitative PCR for gene expression analysis**. *BioTechniques* 2008, **44**:619-626.

19. Andersen C, Jensen JL, Orntoft TF: **Normalization of real-time quantitative reverse transcription-PCR data: a model-based variance estimation approach to identify genes suited for normalization, applied to bladder and colon cancer data sets**. *Cancer Res* 2004 **64**:5245-5250.

20. Vandesompele J, De Preter K, Pattyn F, Poppe B, Van Roy N, De Paepe A, Speleman F: **Accurate normalization of real-time quantitative RT-PCR data by geometric averaging of multiple internal control genes**. *Genome Biol* 2002, **3**:R0034.

21. Jafari Anarkooli I, Sankian M, Ahmadpour S, Varasteh AR, Haghir H: **Evaluation of Bcl-2 family gene expression and caspase-3 activity in hippocampus of STZ-induced diabetic rats**. *Exp Diabetes Res* 2008, **2008:638467**.

22. Mane VP, Heuer MA, Hillyer P, Navarro MB, Rabin RL: **Systematic method for determining an ideal housekeeping gene for real-time PCR analysis**. *J Biomol Tech* 2008, **19**:342-347.
